# Inflammatory biomarkers profiles and cognition among older adults

**DOI:** 10.1038/s41598-025-86309-z

**Published:** 2025-01-17

**Authors:** Aline Thomas, Jing Guo, Dolly Reyes-Dumeyer, Danurys Sanchez, Nikolaos Scarmeas, Jennifer J. Manly, Adam M. Brickman, Rafael A. Lantigua, Richard Mayeux, Yian Gu

**Affiliations:** 1 Taub Institute for Research On Alzheimer’s Disease and the Aging Brain, Vagelos College of Physicians and Surgeons, Columbia University, New York, NY 10032 USA; 2 G.H. Sergievsky Center, Vagelos College of Physicians and Surgeons, Columbia University, New York, NY 10032 USA; 3 Department of Neurology, Vagelos College of Physicians and Surgeons, Columbia University, and the New York Presbyterian Hospital, New York, NY 10032 USA; 4 1st Department of Neurology, Aiginition Hospital, National and Kapodistrian University of Athens Medical School, Athens, Greece; 5 Department of Medicine, Vagelos College of Physicians and Surgeons, Columbia University, and the New York Presbyterian Hospital, New York, NY 10032 USA; 6 Department of Epidemiology, Mailman School of Public Health, Columbia University, New York, NY 10032 USA; 7 Departments of Neurology and Epidemiology, Taub Institute, and Sergievsky Center, Columbia University Irving Medical Center, 622 W 168Th St., New York, NY 10032 USA

**Keywords:** Peripheral inflammation, Cognition, Cognitive decline, Cohort, Epidemiology, Epidemiology, Inflammation, Cognitive ageing

## Abstract

Inflammation plays a major role in cognitive aging. Most studies on peripheral inflammation and cognitive aging focused on selected major inflammatory biomarkers. However, inflammatory markers are regulated and influenced by each other, and it is therefore important to consider a more comprehensive panel of markers to better capture diverse immune pathways and characterize the overall inflammatory profile of individuals. We explored 23 circulating inflammatory biomarkers using data from 1,743 participants without dementia (≥ 65 years-old) from the community-based, multiethnic Washington Heights Inwood Columbia Aging Project. Using principal component analysis (PCA), we developed six inflammatory profiles (PC-1 to PC-6) based on these 23 biomarkers and tested the association of resulting inflammatory profile with cognitive decline, over up to 12 years of follow-up. PC-1 described a pro-inflammatory profile characterized by high positive loadings for pro-inflammatory biomarkers. A higher PC-1 score was associated with lower baseline cognitive performances. No association of this profile with cognitive decline was observed in longitudinal analysis. However, PC-5 characterized by high PDGF-AA and RANTES was associated with a faster cognitive decline. Among older adults, a circulating pro-inflammatory immune profile is associated with lower baseline cognitive performance, and some specific pro-inflammatory cytokines might be associated with faster cognitive decline.

## Introduction

Inflammation plays a major role in the development of Alzheimer’s disease (AD) dementia and cognitive aging in general^1^. Inflammation is part of brain defense mechanism against stressors, with microglia activation triggering the release or attraction of inflammatory mediators such as cytokines and free radicals to maintain homeostasis and ensure healthy neural functions. However, in the aging brain, prolonged chronic over-activation of microglia can become neurotoxic; microglia activation together with β-amyloid and tau pathologies leads to an exacerbated release of pro- and anti-inflammatory proteins, which might trigger neurodegeneration and blood brain barrier leakage, and in turn associated cognitive impairment^2^. Moreover, inflammatory markers can also promote the accumulation of AD pathology, which increase under inflammatory conditions^3,4^. Thus, the complex relationship of inflammation with brain aging can include both protective and harmful roles, and it is still unclear whether neuroinflammation is caused by or leads to cognitive decline^5^.

In addition to neuroinflammation, systemic inflammation, including peripheral inflammatory mediators, is also involved in neurodegeneration and AD pathogenesis^6,7^. Cytokines and inflammatory markers can cross the blood brain barrier through multiple pathways, making it feasible for circulating cytokines to enter the brain^8–10^. Circulating levels of cytokines are associated with AD diagnosis, cognitive performances, and brain structure in several studies, although mixed results have also been reported^11–16^. Most studies on circulating inflammatory biomarkers and brain aging outcomes investigated clinical diagnosis of dementia, rather than more subtle cognitive decline, and focused on one or a few major inflammatory biomarkers, mostly pro-inflammatory, such as C-Reactive Protein (CRP), IL-6, or Tumor Necrosis Factor α (TNF-α)^17^. However, inflammatory markers are regulated and influenced by each other, and it is therefore important to consider a more comprehensive panel of inflammatory markers to better capture the overall inflammatory profile of individuals. For example, a study found ten inflammatory markers as a group were associated with total brain volume (TBV), but only three of those ten were individually associated with TBV^18^. In another study, chemokine-cytokine pattern of sIL4R, IL6, and IL8, but not individual cytokine levels, was associated with brain atrophy^19^. Similarly, a combination of ten inflammation biomarkers had a stronger association with cognitive decline than individual biomarkers^20^. Thus, to better characterize the role of systemic inflammation in cognitive aging, it is important to examine a large panel of circulating inflammatory markers simultaneously, characterizing diverse immune pathways and their association with long term cognitive decline^21,22^. To this end, principal component analysis (PCA), a widely used tool for dimensionality reduction, can be used to reduce many interrelated variables into fewer, more meaningful ones (i.e. latent components) while identifying the relative importance of each marker^23^.

In the present study, we explored 23 circulating inflammatory biomarkers using data from a community-based, multiethnic cohort of elderly participants without dementia, the Washington Heights/Hamilton Heights Inwood Columbia Aging Project (WHICAP). We developed inflammatory profiles based on these 23 blood inflammatory biomarkers and tested the association of resulting inflammatory biomarkers profiles with cognitive decline.

## Methods

### Study population

WHICAP is an ongoing community-based study of aging and dementia including participants identified from a probability sample of Medicare beneficiaries residing in northern Manhattan^24^. Participants, older than 65 years and fluent in English and/or Spanish, were recruited in 3 waves, starting in 1992, 1999, and 2009^25,26^. Participants completed a baseline assessment and were followed up every 18 to 24 months. At each visit, information about general health, medical and neurological histories were collected, functional ability was assessed, and a neuropsychological battery was administrated. Additionally, blood samples were collected at baseline. Diagnosis of dementia was based on standard research criteria using all available information at a consensus conference^27^.

The current analysis includes participants from the 2009 cohort (n = 2,425) for which inflammatory biomarkers were measured at baseline. We excluded participants with dementia at baseline (n = 159), without inflammatory markers’ measures or with missing data for one or more inflammatory biomarkers (n = 364), and with missing data for cognition (n = 159), leaving 1,743 participants included in the analysis (Supplementary Fig. 1).

The study protocol and all methods were approved by the Institutional Review Boards at Columbia University in accordance with the Declaration of Helsinki. All participants provided written informed consent.

### Inflammatory biomarkers quantification

The circulating levels of inflammatory biomarkers, including interleukins, chemokines, interferons, growth factors, colony stimulating factors, and others were measured from blood collected at baseline. Blood samples were collected in BD Vacutainer Ethylenediaminetetraacetic acid (EDTA) tubes and centrifuged at 2000 g for 15 min at room temperature and then stored at − 80 °C in polypropylene cryotubes until analysis. We selected a panel of 23 inflammatory biomarkers based on biological functions^28^, role in AD pathogenesis^29^, value in AD diagnosis and prognosis^21^, relationship with structural/functional neuroimaging findings^15,30^, and their genetic polymorphisms in susceptibility of AD^31^, availability of commercial assay kits, and capacity to be measured within detection limits (detected in at least 60% samples) and to displayed fair within-person temporal reproducibility (intraclass correlation coefficients > 0.40), as identified in a previous study^32,33^. Biomarkers were measured by the Luminex technology (MILLIPLEX MAP Human Cytokine/Chemokine Magnetic Bead Panel [HCYTOMAG-60 K] and High Sensitivity T Cell Panel [HSTCMAG-28SK]), except for CRP which was analyzed by the Integra technology. Analyses were performed according to manufacturer instructions. Samples with biomarker levels less than the lower limit of detection (LLOD) were assigned a value of LLOD divided by the square root of two, and samples with levels beyond the upper limit of detection (ULOD) were assigned with the ULOD value^32^. The 23 biomarkers meeting the above criteria to be retained for analysis integrated various immune response pathways and functions: 15 pro-inflammatory markers (CRP, Eotaxin, Growth Regulated Oncogene chemokine [GRO], IL-1β, IL-6, IL-7, IL-12p70, IL-17A, Inducible Protein 10 [IP-10], Monocyte Chemoattractant Protein 1 [MCP-1], Macrophage Inflammatory Protein 1β [MIP-1β], Platelet Derived Growth Factor AA [PDGF-AA], Regulated upon Activation Normal T-cell Expressed and Secreted [RANTES], TNF-α, and Vascular Endothelial Growth Factor [VEGF]); 5 anti-inflammatory markers (IL-1 Receptor Antagonist [IL-1RA], IL-4, IL-10, IL-13, and Macrophage Derived Chemokine [MDC]); and 3 markers that had mixed pro- and anti-inflammation properties (Fractalkine, Granulocyte/Macrophage Colony-Stimulating Factor [GM-CSF], Transforming Growth Factor α [TGF-α]). For analysis, inflammatory biomarkers’ measures were log-transformed and standardized into z-scores.

### Cognitive assessment

Cognitive performance was assessed with a neuropsychological battery, administrated in English or Spanish at baseline and each follow-up visit^25,34,35^. Participants had between 1 and 6 assessments over the 12-year follow-up (n = 387 had 1 assessment, n = 352 had 2, n = 406 had 3, n = 448 had 4, n = 141 had 5, and n = 9 had 6 assessments). The performances in four cognitive domains (memory, language, processing speed, and visuospatial ability) were captured by neuropsychological tests which were determined and selected according to a previously published confirmatory factor analysis^35^. Memory was assessed with the immediate and delayed recalls from the Selective Reminding Test^36^ and the recognition component of the Benton Visual Retention Test^37^. Language score combined naming (15-item Boston Naming Test)^38^, letter fluency (Controlled Word Association)^39^, category fluency (Boston Diagnostic Aphasia Examination)^40^, verbal abstract reasoning (Similarities subtest of the Wechsler Adult Intelligence Scale–Revised)^41^, and repetition and comprehension (Boston Diagnostic Aphasia Examination)^40^. Processing speed was computed using the Color Trails Test 1 and 2. Visuospatial function was assessed using the Rosen Drawing Test^42^, the matching component from the Benton Visual Retention Test^37^, and the Identities and Oddities subset of the Mattis Dementia Rating Scale^43^.

Individual neuropsychological tests were standardized into z-scores using baseline means and SDs. Domain-specific scores were then computed as the mean of the individual test z-scores. The four resulting domain z-scores were averaged to create a composite global cognition z-score. Higher z-scores indicate better cognitive performance.

## Covariates

Sociodemographic and lifestyle variables were derived from baseline evaluation and included age, gender, race/ethnicity (non-Hispanic Black, Hispanic, non-Hispanic White, or other based on self-report using the format of the 2000 US Census)^44^, years of education, body mass index (in kg/m^2^), and smoking status (never, former, current). Apolipoprotein E (*APOE*) genotype was considered dichotomously (no ɛ4 allele versus carrying at least one ɛ4 allele). Presence or absence of heart disease, diabetes mellitus, and hypertension were based on self-report information, and clinical stroke was determined by self-reports supplemented by neurological examination or review of medical records. These four dichotomized vascular comorbidities were summed into a vascular comorbidity burden score (range, 0–4). Use of anti-inflammatory medication including aspirin and other non-steroidal anti-inflammatory drugs was self-reported.

### Statistical analysis

#### Construction of inflammatory biomarker profiles

We identified underlying latent inflammatory profiles in our population as the combination of 23 inflammatory markers by principal component analysis (PCA) with orthogonal Varimax rotation^45^. PCA aims to reduce the dimension of a dataset from multiple variables to a few uncorrelated summary variables, defined as linear combination of predictors, that explain as much as possible of the variance of the data; thus, increasing the interpretability of the data while preserving most of the information. The Varimax-rotated PCA was performed using the *principal()* function from the ‘psych’ R package. The number of profiles to be retained was determined by screeplot and the Kaiser Criterion (Supplementary Fig. 2). Individual PC scores were computed as the linear combination of all 23 biomarkers weighted by the rotated PCA component coefficients. PC scores were categorized into tertiles for descriptive and visual representation purposes but were used as a continuous variable for analyses.

#### Demographic and clinical characteristics of the study population

Demographic and clinical characteristics were described across the tertiles of inflammatory profile scores, using means and standard deviations (SD) for quantitative variables and percentages for categorical variables. Differences of characteristic across tertiles of inflammatory profile score were tested with chi-square test for categorical variables and ANOVA for continuous variables.

#### Association between inflammatory biomarker profiles and cognition

The association between inflammatory biomarker profiles and cognitive decline were estimated by linear mixed models with random intercept and slope. The models included an intercept representing cognitive scores at baseline, continuous inflammatory profile score, time of follow-up (with corresponding correlated individual random effects to evaluate individual linear cognitive trajectory), an interaction of inflammatory profile score with time, covariates (age, gender, race/ethnicity, *APOE* ɛ4, and indicator for the first cognitive assessment), and their interaction covariates with time. A significant interaction between inflammatory profile score and time would indicate a different rate in cognitive trajectory over time by inflammatory profile score.

### Supplementary analysis

We performed a series of sensitivity analyses. First, in addition to the covariates in Model 1 (age, gender, race/ethnicity, *APOE* ɛ4, and indicator for the first cognitive assessment to account for retest effect^46^), models were adjusted for education (a proxy for socio-economic status; Model 2), for vascular risk factors associated with both inflammation status and risk of cognitive decline (body mass index, smoking status, vascular comorbidity score; Model 3), and for all the above covariates and the use of anti-inflammatory medication (Model 4). Second, we tested effect modification by age, gender, race/ethnicity, and *APOE* ɛ4 status. Third, we explored non-linear trajectories for cognitive decline by using natural cubic splines for the time variable in the linear mixed model. Finally, we explored all 23 inflammatory biomarkers for their individual relationship with cognition. In this last analysis, adjustment for multiple comparison was performed by using False Discovery Rate correction.

## Results

### Inflammatory profiles

Most of the variance in participants’ inflammatory markers’ levels was summarized by the first six PCA components (explained variances: 17.1%, 16.3%, 14.0%, 6.1%, 6.0%, and 5.5%, respectively) (Supplementary Fig. 2 and Table 1). The first rotated PCA component (PC-1) explained the most variances of the 23 inflammatory biomarkers, it had positive loadings for 20 of them and displayed a meaningful interpretation in terms of inflammation profiles (Table 1). Thus, in the subsequent analyses, we focused on this PC-1 and analyses for the other five PCs are presented in supplementary analyses.

**Table 1:** Coefficients of the 23 inflammatory biomarkers for the first 6 components of the rotated Principal Component Analysis, WHICAP (n = 1,743).

Inflammatory functions	Biomarker	PC-1	PC-2	PC-3	PC-4	PC-5	PC-6
Pro-inflammatory	MIP-1β	**0.80**	− 0.03	− 0.04	0.13	0.10	0.04
	IL-1β	**0.79**	**0.37**	0.19	0.08	0.05	− 0.18
	TNF-α	**0.77**	0.02	0.08	0.03	0.07	**0.28**
	MCP-1	**0.67**	− 0.001	− 0.07	** − 0.31**	0.18	− 0.12
	IL-6	**0.65**	0.04	**0.63**	0.09	0.02	− 0.10
	IP-10	**0.34**	0.06	0.01	− 0.17	** − 0.42**	**0.38**
	VEGF	**0.32**	0.19	**0.25**	− 0.003	**0.29**	**0.40**
	RANTES	**0.25**	− 0.04	− 0.06	− 0.01	**0.53**	− 0.02
	Eotaxin	**0.20**	− 0.01	− 0.02	** − 0.71**	**0.25**	0.19
	GRO	0.10	− 0.02	− 0.10	**0.72**	**0.24**	0.09
	IL-12p70	0.10	**0.87**	0.16	− 0.01	− 0.04	0.01
	CRP	0.09	− 0.01	− 0.04	**0.43**	− 0.02	**0.35**
	PDGF-AA	0.08	− 0.09	0.01	− 0.03	**0.78**	0.09
	IL-17A	0.02	**0.86**	0.18	− 0.03	− 0.06	0.02
	IL-7	− 0.10	**0.78**	0.14	− 0.01	0.07	0.06
Pro- and anti-inflammatory	TGF-α	**0.35**	0.18	**0.49**	− 0.08	0.12	**0.39**
	GM-CSF	**0.29**	**0.71**	0.16	0.07	− 0.09	− 0.16
	Fractalkine	0.11	**0.82**	0.11	− 0.05	− 0.08	0.03
Anti-inflammatory	IL-1RA	**0.78**	0.13	0.19	− 0.05	0.07	0.13
	IL-10	0.14	**0.34**	**0.82**	− 0.03	− 0.04	0.003
	IL-13	0.03	**0.30**	**0.87**	− 0.05	− 0.05	− 0.02
	IL-4	− 0.01	0.10	**0.93**	− 0.06	− 0.04	− 0.03
	MDC	− 0.06	− 0.08	− 0.07	0.07	0.003	**0.68**

Principal Component (PC) scores were computed as the linear combination of the 23 inflammatory biomarkers weighted by rotated PCA coefficients. PC-1 was used for the main analysis. It explained 17.1% of the variance of all biomarkers and identified a pro-inflammatory profile with positive loadings for 20 out of 23 markers and specifically high loadings for pro-inflammatory biomarkers (e.g., MIP-1β, IL-1β, TNF-α, MCP-1, and IL-6). Markers with moderate to high contribution to PCs (coefficients >|0.20|) are indicated in bold. PC-2 explained 16.3% of the variance of all biomarkers; PC-3, 14.0%; PC-4, 6.1%; PC-5, 6.0%; and PC-6, 5.5%. Inflammatory biomarkers are classified based on their main functions reported in the literature: pro-inflammatory, anti-inflammatory, or both pro- and anti-inflammatory for biomarkers exhibiting both properties depending on the context of their expression and the cellular environment.

PC-1 was predominantly defined by high positive loadings (≥ 0.30) for MIP-1β, IL-1β, IL-1RA, TNF-α, MCP-1, IL-6, TGF-α, IP-10, and VEGF. All but one (IL-7) pro-inflammatory markers had positive loadings for PC-1, with moderate (0.20 to 0.30) positive loadings for GM-CSF, RANTES, and Eotaxin. Four (IL-10, IL-4, IL-13, and MDC) of five anti-inflammatory markers had negative and/or low loadings (-0.06 to 0.14) (Table 1). Thus, PC-1 score could be described as generally pro-inflammatory. A higher PC-1 score indicates a more pro-inflammatory profile.

### Characteristics of the study population

Among the 1,743 participants included, the majority were women (67%), and the mean age was 74.2 (± 6.1) years (Table 2). Compared to participants with more anti-inflammatory biomarker profile (i.e., low PC-1 score), participants with more pro-inflammatory profile were older, more likely to be Hispanic, and had lower education level. They also tended to have higher BMI, higher vascular comorbidity scores, and took more anti-inflammatory medications.

**Table 2:** Participants’ characteristics, WHICAP (n = 1,743).

	Total Population	PC-1 score categories			
		T1 (n = 581)	T2 (n = 581)	T3 (n = 581)	*P*-values
Age, mean (SD)	74.2 (6.1)	72.3 (5.4)	73.9 (5.9)	76.4 (6.3)	< 0.001
Female, n (%)	1161 (66.6)	397 (68.3)	388 (66.8)	376 (64.7)	0.42
Education (years), mean (SD)	11.3 (5.1)	12.6 (4.6)	11.6 (5.1)	9.7 (5.1)	< 0.001
Race/Ethnicity, n (%)					< 0.001
Hispanic	839 (48.1)	209 (36.0)	264 (45.4)	366 (63.0)	
Non-Hispanic Black	478 (27.4)	194 (33.4)	167 (28.7)	117 (20.1)	
Non-Hispanic White	396 (22.7)	167 (28.7)	134 (23.1)	95 (16.4)	
Others	30 (1.7)	11 (1.9)	16 (2.8)	3 (0.5)	
*APOE* ɛ4 carrier, n (%)	458 (26.3)	154 (26.5)	168 (28.9)	136 (23.4)	0.10
BMI (kg/m^2^), mean (SD)	28.0 (4.4)	27.5 (4.4)	28.1 (4.3)	28.6 (4.5)	< 0.001
Smoking status, n (%)					0.02
Never	910 (52.2)	299 (51.5)	283 (48.7)	328 (56.5)	
Former	702 (40.3)	229 (39.4)	261 (44.9)	212 (36.5)	
Current	131 (7.5)	53 (9.1)	37 (6.4)	41 (7.1)	
Diabetes, n (%)	584 (33.5)	172 (29.6)	195 (33.6)	217 (37.3)	0.02
Hypertension, n (%)	1461 (83.8)	467 (80.4)	483 (83.1)	511 (88.0)	0.002
Stroke, n (%)	91 (5.3)	26 (4.5)	32 (5.6)	33 (5.7)	0.61
Heart diseases, n (%)	656 (37.6)	199 (34.3)	221 (38.0)	236 (40.6)	0.08
Vascular comorbidity score, mean (SD)	1.60 (0.95)	1.49 (0.96)	1.60 (0.99)	1.72 (0.89)	< 0.001
Anti-inflammatory medication, n (%)	790 (45.3)	225 (38.7)	270 (46.5)	295 (50.8)	< 0.001
Global cognition composite score (range, -3.5; 1.7), mean (SD)	0.00 (0.84)	0.27 (0.75)	0.03 (0.84)	− 0.29 (0.82)	< 0.001
Memory composite score (range, -2.3; 2.0), mean (SD)	0.36 (0.74)	0.50 (0.74)	0.36 (0.77)	0.20 (0.68)	< 0.001
Processing speed composite score (range, -4.0; 2.0), mean (SD)	0.28 (1.11)	0.61 (0.87)	0.34 (1.09)	− 0.10 (1.23)	< 0.001
Visuospatial functions composite score (range, -2.3; 1.4), mean (SD)	0.42 (0.58)	0.60 (0.51)	0.42 (0.58)	0.23 (0.58)	< 0.001
Language composite score (range, -2.1; 3.3), mean (SD)	0.43 (0.67)	0.61 (0.64)	0.46 (0.68)	0.23 (0.64)	< 0.001

*APOE*ɛ4, ɛ4 allele of the apolipoprotein E gene; BMI, body mass index; PC-1, principal component 1; SD, standard deviation; T1-3, tertiles 1 to 3.

Categories of PC-1 pro-inflammatory score are defined by tertiles of score, from least pro-inflammatory (T1) to most pro-inflammatory (T3). Means and percentages are of non-missing values. Missing values: 12.3% for BMI, 0.6% for stroke and vascular comorbidities score, and 0.3% for education level. *P*-values estimates by chi-square test for categorical variables and ANOVA for continuous variables.

### Inflammatory biomarker profile and cognition

Cognitive status was evaluated at baseline and over follow-up for a maximum of 12.5 years (median = 4.6 years). A higher PC-1 pro-inflammatory score was associated with worse cognitive performances at baseline for global cognition, processing speed, visuospatial functions, and language, but not memory (Table 3). Each 1-SD increase in PC-1 score was associated with a decrease of 0.05 SD (95% CI, -0.09; -0.02, p = 0.004) in global cognitive score, which was equivalent to the cognitive effect estimate for a 1-year increase in age at baseline (β = -0.05 [-0.06; -0.05], p < 0.001). Similarly, the effect estimates were β = -0.07 SD (-0.11; -0.03, p < 0.001) for processing speed, β = -0.06 (-0.10; -0.03, p = 0.001) for visuospatial functions, and β = -0.04 SD (-0.08; -0.001, p = 0.04) for language score. Longitudinally, PC-1 pro-inflammatory score was not associated with cognitive decline in any cognitive domains (all p-values for PC-1 score-by-time interaction terms > 0.05; Table 3).

**Table 3:** Associations of PC-1 and PC-5 scores with cognition, estimated by linear mixed models, WHICAP (n = 1,743).

	Baseline cognitive performances		Cognitive change	
	β [95%CI]	P-value	β [95%CI]	P-value
	PC-1 score			
Global cognition	− 0.05 [− 0.09; − 0.02]	0.004	0.001 [− 0.004; 0.01]	0.71
Memory	− 0.01 [− 0.05; 0.03]	0.69	0.0003 [− 0.01; 0.01]	0.94
Processing speed	− 0.07 [− 0.11; − 0.03]	< 0.001	− 0.001 [− 0.007; 0.01]	0.82
Visuospatial function	− 0.06 [− 0.10; − 0.03]	0.001	0.004 [− 0.003; 0.01]	0.25
Language	− 0.04 [− 0.08; − 0.001]	0.04	0.003 [− 0.002; 0.01]	0.25
	PC − 5 score			
Global cognition	− 0.03 [− 0.06; 0.01]	0.14	− 0.01 [− 0.01; − 0.002]	0.004
Memory	− 0.02 [− 0.06; 0.02]	0.43	− 0.01 [− 0.02; − 0.003]	0.01
Processing speed	− 0.03 [− 0.07; 0.005]	0.09	− 0.002 [− 0.01; 0.004]	0.54
Visuospatial function	− 0.01 [− 0.05; 0.03]	0.55	− 0.01 [− 0.02; − 0.003]	0.01
Language	− 0.03 [− 0.06; 0.01]	0.16	− 0.001 [− 0.01; 0.004]	0.70

The table presents the β coefficients and 95% confidence intervals for the associations of continuous PC-1 and PC-5 scores with cognitive z-scores at baseline and cognitive decline over the 12-year follow-up. The trajectories of change in cognition were estimated using linear mixed models across up to 6 repeated neurocognitive examinations. Models consider a linear function of time, with corresponding random effect, and include an intercept representing the cognitive z-score at baseline (and corresponding random effect), PC-1 pro-inflammatory score or PC-5 score (continuous, standardized), covariates (age, gender, race/ethnicity, status for ɛ4 allele of the apolipoprotein E [*APOE*ɛ4] gene, and indicator for first cognitive assessment), and their interactions with time. The estimate for baseline association is the coefficient for the PC-1 pro-inflammatory score or PC-5 variable term; and the estimate for cognitive decline is the coefficient for the PC-1 or PC-5 score-by-time interaction term. Effect estimates are reported for 1-SD increase in PC scores.

### Sensitivity analyses

The associations of higher PC-1 pro-inflammatory score with lower cognition at baseline were robust to adjustment for vascular risk factors and use of anti-inflammatory medication (Supplementary Fig. 3). There was an association with baseline processing speed in all models (β = -0.05 SD [-0.09; -0.01] in fully adjusted model), but associations with global cognition, visuospatial function, and language were attenuated and no longer significant after adjustment for education.

Testing the interactions of PC-1 score with age, gender, race/ethnicity, and *APOE*ɛ4 status on baseline cognition, we found moderation effects by gender for global cognition and processing speed (p for interaction < 0.04), by race/ethnicity for language (p for interactions < 0.03), and by *APOE* ɛ4 status for visuospatial functions (p for interaction = 0.04). In stratified analyses, PC-1 scores were associated with baseline performances in global cognition, processing speed, and language among women only (Supplementary Fig. 4); with lower scores in global cognition, processing speed, and visuospatial functions for Hispanic participants only, with language for Non-Hispanic White participants only, but no association was observed for Non-Hispanic Black participants (Supplementary Fig. 5). For *APOE* ɛ4 status, stronger effects were observed for ε4 carriers compared with non-carriers, although baseline associations with global cognition, processing speed, and visuospatial functions remained significant for both groups (Supplementary Fig. 6).

Considering nonlinear trajectories of cognitive decline with time approximated by natural cubic splines (two internal knots placed at tertiles of measurements times) did not modify the results and the cognitive trajectories were not significantly different by PC-1 score.

### Other inflammatory biomarker profiles and cognition

Analyses for PCs 2 to 6 are presented in Supplementary Fig. 7. None of the PC scores were associated with cognitive performances at baseline, but higher PC-5 score was associated with greater cognitive decline in global cognition, memory, and visuospatial function (β = -0.01, 95%CI = [-0.01; -0.002]; -0.01 [-0.02; -0.003]; and -0.01 [-0.02; -0.003], respectively) (Table 3). PC-5 was characterized by high-to-moderate positive loadings for several pro-inflammatory markers (PDGF-AA [0.78], RANTES [0.53], VEGF [0.29], Eotaxin [0.25], and GRO [0.24]) and negative loading (-0.42) for only one pro-inflammatory marker IP-10, while none of the anti-inflammatory or mixed function biomarkers played key roles in PC-5 (Table 1).

### Exploratory analyses on individual inflammatory biomarkers and cognition

**Fig. 1:**
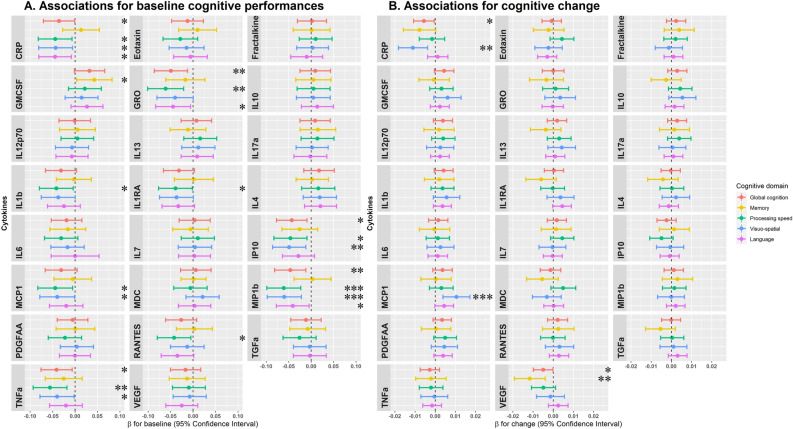
Associations of 23 inflammatory biomarkers with cognition, estimated by linear mixed models, WHICAP (n = 1743). The figure displays the effect estimates for the associations of continuous inflammatory biomarkers with cognitive z-scores at baseline (Panel A) and cognitive decline (Panel B) over the 12-year follow-up. The trajectories of change in cognition were estimated using linear mixed models across up to 6 repeated neurocognitive examinations. Models consider a linear function of time, with corresponding random effect, and include an intercept representing the cognitive z-score at baseline (and corresponding random effect), inflammatory markers (continuous, log-transformed and standardized), covariates (age, gender, race/ethnicity, status for ɛ4 allele of the apolipoprotein E gene, and indicator for first cognitive assessment), and their interactions with time. The estimate for baseline association (Panel A) is the coefficient for the inflammatory markers variable term; and the estimate for cognitive decline (Panel B) is the coefficient for the biomarker-by-time interaction term. Effect estimates (β coefficients and 95% confidence intervals) are reported for 1-SD increase in log-transformed biomarker value. Significant associations are indicated by an asterisk: * for p-value < 0.05, ** for p-value < 0.01, and *** for p-value < 0.05 after False Discovery Rate correction for multiple testing.

Results of the associations between the 23 individual inflammatory biomarkers and baseline cognitive performances are presented in Fig. 1A. A higher circulating level of CRP, a well-known pro-inflammatory marker, was associated with lower scores in global cognition, processing speed, visuospatial functions, and language. Moreover, for three pro-inflammatory biomarkers with high positive loading on PC-1 (i.e. MIP-1β [FRD-corrected p < 0.05], TNF-α, and MCP-1), as well as GRO and IP-10, higher levels were associated with lower baseline cognition. GM-CSF, which had high positive loading on PC-1 was associated with higher baseline memory. IL-1RA, an anti-inflammatory marker with high positive loading on PC-1 (0.78), also showed associations with lower baseline cognition.

For cognitive change over follow-up, higher levels of CRP were associated with faster decline in global cognition and visuospatial function. Higher levels of VEGF were associated with faster decline in global cognition and memory. Higher MCP-1 levels were associated with slower decline in visuospatial functions (FDR-corrected p < 0.05) (Fig. 1B).

Only associations of MIP-1β with baseline visuospatial and processing speed performances, and of MCP-1 with visuospatial decline remained significant after FDR correction.

## Discussion

We analyzed data from the large multi-ethnic population-based WHICAP cohort to evaluate the relationship of 23 circulating inflammatory biomarkers with cognition. We found that a higher PCA-defined pro-inflammatory biomarker profile score, and higher circulating levels of its key drivers (MIP-1β, TNF-α, and MCP-1) were associated with lower baseline cognitive performances in global cognition, visuospatial functions, processing speed, and language. The effect size observed for a 1-SD increase in PC-1 pro-inflammatory profile was equivalent to approximately 1 year of aging. The associations were robust to adjustment for demographic characteristics, vascular risk factors and use of anti-inflammatory medication, and attenuated by adjustment for education. We found the pro-inflammatory PC-1 was not associated with cognitive decline over 12 years of follow-up. However, another pro-inflammatory PC-5, characterized by high levels of PDGF-AA for vascular permeability and RANTES for regulating immune cell migration^47,48^, and individual markers VEGF and CRP were associated with a faster decline in global cognition.

There is substantial evidence that peripheral inflammation plays a role in cognitive aging^1,2,8–10^. However, the relationship is complex, with both protective and detrimental roles of inflammation, and an unclear directionality as to whether neuroinflammation is caused by or leads to cognitive decline^5^. Our results reflect previous findings of associations of elevated levels of key inflammatory factors, such as CRP or TNF-α, and lower cognitive functions^13,17,49–51^.

There are several biological pathways that could potentially explain the association of these inflammatory markers with cognition. CRP is associated with changes in the expression of adhesion molecules promoting disruption of the endothelium and development of atherosclerosis, leading to vascular dysfunction and neuronal damage^52,53^. We also found association with cognition in VEGF as well as a few less studied chemokines (MCP-1, MIP-1β, IP-10, GRO, GM-CSF, and RANTES), which play an important role in recruiting microglia, astrocytes, and other immune cells. VEGF plays a major role in vascular permeability and blood–brain barrier integrity by regulating blood vessel growth and astrocytes migration^54^. MCP-1 regulates inflammatory processes by modulating endothelial activation and monocyte chemotaxis^55^. MIP-1β has a coordinating role in the immune response by acting as a chemoattractant for multiple immune cells, including macrophages, T cells, and dendritic cells^56^. IP-10 acts as a chemoattractant for activated T cells^57^. GRO is involved in the recruitment and activation of neutrophils^58^. RANTES is highly expressed in microglia and is involved in immune response but also neurotransmission and neuroplasticity^59^.

Beyond well studied CRP, IL-6 and TNF-α, literature on other chemokines is sparser and mainly focus on clinically defined AD or mild cognitive impairment (MCI). The current literature on inflammatory biomarkers and cognition has been inconsistent, with some studies suggesting that the associations of chemokines with cognition might differ according to disease stage. For example, higher levels of circulating MCP-1 have been observed in participants with AD compared to participants with MCI and healthy controls, and in severe AD compared to mild-to-moderate AD. In this same study of 496 participants recruited from outpatient general clinics, higher MCP-1 was associated with faster decline in MMSE^60^. While a study of 720 participants of the AddNeuroMed study reported higher MCP-1 levels in MCI participants compared to healthy control or AD participants^61^. In contrast, another study of 290 women reported lower levels of serum MCP-1 in MCI compared to health controls, but no difference of serum MCP-1 between AD and healthy controls^62^, and another did not find any association (n = 472)^63^. Other studies with smaller sample sizes also reported mixed results^12,64^. Similar inconsistent results were reported for other inflammatory markers. For example, lower levels of MIP-1β, RANTES, and VEGF were observed in moderate AD compared to controls in one small case–control study (n = 50)^64^. In another study of 290 elderly women, while there were lower levels of MIP-1β in participants with MCI compared to controls, there were higher levels of MIP-1β, GRO, and IP-10 in participants with dementia relative to controls^62^. However, these studies were relatively small, with cross-sectional or case–control design, and evaluated clinical diagnosis rather than cognitive performance and decline. The inconsistent results might be due to differences in population demographics (e.g. population-based cohort or patients from memory clinics), disease ascertainment process or stage, or cytokine measurements. Other cytokines show promising results treatment trials. For example, sargramostim, a recombinant human GM-CSF, has been found to be efficacious in animal and human studies of AD^65^. Thus, our study provides important evidence and support to investigate novel inflammatory biomarkers in future pharmaceutical interventions.

Overall, the literature on individual inflammatory biomarkers and dementia status or cognition reports conflicting associations and is limited for older adults without dementia. Our results add to previous research on associations of specific markers of inflammation with cognitive outcomes in clinically healthy older adults and go further by examining a more comprehensive selection of inflammatory markers. The described associations of several individual biomarkers with cognitive performances suggest the involvement of multiple immune pathways. As cytokines are regulated and influenced by each other, approaches combining biomarkers that affect multiple pathways simultaneously, such as our PCA-defined pro-inflammatory profile, may better reflect overall inflammatory patterns of an individual and better inform on the role of inflammation in cognitive aging.

As previously described, the close link between peripheral inflammation and brain aging is largely agreed upon, but the relationship between the two processes is complex and the direction not straightforward. On the one hand, circulating inflammatory biomarkers may contribute to neurotoxicity by crossing and increasing trafficking across the blood–brain barrier^8–10^. Inflammatory markers interacting with the central nervous system can influence brain aging, either directly or by triggering activation of the microglia and astrocytes to release pro-inflammatory molecules. On the other hand, the increase of circulating biomarkers of inflammation can be the consequence of the release of cytokines into the peripheral following inflammatory responses to early brain pathology. Our results, showing an association of baseline pro-inflammatory biomarker profile PC-1 and its key components with baseline cognitive performance but an absence of longitudinal association with cognitive decline over follow-up, is more consistent with the latter possibility. However, we also found PC-5, with major components including VEGF and PDGF-AA^47^, both involved in vascular permeability, and RANTES^48^, a chemokine that regulates immune cell migration, to be related with cognitive decline, supporting the first pathway. Therefore, the exact pathways might differ depending on the inflammatory marker involved. For example, in our study, several key contributors for PC-1 (MIP-1β, TNF-α, MCP-1, IP-10, IL-1RA, GM-CSF) were significantly associated baseline cognition but not cognitive decline, suggesting these inflammatory biomarkers either play important roles in accumulative chronic systemic inflammation or are responsive to early pathological changes, or both. In contrast, VEGF, a major component of PC-5 showed association with cognitive decline and thus may contribute to the development of AD. Future longitudinal studies including repeated measurement of biomarkers over time and examination of inflammatory biomarkers with brain pathology would improve our understanding of the temporality and directionality of the association of peripheral inflammation with cognitive aging.

Our study has several strengths, including a large population-based sample with longitudinal assessment of cognition over 12 years. Moreover, our cohort consider multiple ethnic groups, including White but also Black and Hispanic participants, and is thus likely to be more generalizable to the increasingly diverse US population. We identified a stronger association of a pro-inflammatory biomarker profile with worse cognition among Hispanic and Non-Hispanic White participants but not among Non-Hispanic Black participants. These differences among ethnic groups could be due to sample size (Hispanic participants representing 48.1% of the study population) but should be further investigated. We also identified modification effect by *APOE* ɛ4, which had been shown to play an important role in modulating the immune response, either by disruption of lipid transport into the brain inducing chronic inflammation, by impairment of glucose metabolism, or by altering phagocytic abilities of glial cells^2^. Although only significant for visuospatial functions, we found a stronger association among *APOE* ɛ4 carrier than non-carriers, which is consistent with current literature pointing to a stronger association of inflammation with cognition in ε4 carriers^51,66,67^. Further research is needed to fully understand the differential effects of inflammation on cognition based on *APOE* genotype. Clarifying this relationship could have important implications for developing targeted interventions and therapies for individuals at higher risk of cognitive decline due to their *APOE* genotype. Last, our results were robust to adjustment for multiple potential confounders, including use of anti-inflammatory medication, BMI, and vascular risk factor, suggesting that the association of peripheral inflammation with cognitive aging goes beyond the role of inflammation on vascular health^68^.

We acknowledge some limitations. First, our single-time measurement of inflammatory biomarkers may not properly represent the long-term average circulating levels of an individual. However, we selected inflammatory biomarkers with good temporal reproducibility^33^, including among a sample of the participants included in our analysis^32^. Second, although we selected a greater number of biomarkers than most studies, we did not considered some inflammatory markers with poor reproducibility and detectability. Including a larger panel of cytokines might help to better describe the role of inflammation in cognitive aging. Third, the proportion of variance explained by or first PC was modest (17.1%). Previous studies suggest that in healthy older adults, the main principal components can typically capture 50–65% of the total cytokine variations^69,70^. The modest variation explained by the main components in our study may be due to several reasons: (1) the diverse racial/ethnic, educational, lifestyle, and socioeconomic background of our population may lead to an overall larger variance of cytokines than in more homogenous populations; (2) the amount of variation captured by the principal components may decrease with increased number of cytokines included. Thus, although our PCs explained a modest variance of the cytokines, these patterns are still important for their associations with cognition. Fourth, while we adjusted for many potential confounders, including age, vascular disease and use of anti-inflammatory medication, residual confounding from other factors, such as use of other medications like anti-cholinergic drugs^71^ or presence of other chronic conditions, might remain. Last, as mentioned above, we only measured inflammation at baseline. Longitudinal change of markers could potentially help further clarify the temporal relationship between inflammatory biomarkers and cognitive aging.

In our study of multi-ethnic elderly participants, a circulating pro-inflammatory immune profile is associated with lower baseline cognitive performances but not cognitive decline. Our results suggest that the increase of circulating biomarkers of pro-inflammation might be a consequence of the release of inflammatory biomarkers following brain pathology. Studies investigating the longitudinal relationship of circulating inflammation with cognition as well as biomarkers for brain pathology are needed to better understand of the temporality and directionality of the association of peripheral inflammation with cognitive aging.

## Supplementary Information

Supplementary Information.

## Data Availability

The data that support the findings of this study will be made available from the corresponding author Dr. Yian Gu (yg2121@cumc.columbia.edu) upon reasonable request and with permission of the WHICAP Study Committee.
