# Supplementary material for: Inflammatory biomarkers profiles and cognition among older adults
Source: Sci Rep. 2025 Jan 17;15:2265. doi: 10.1038/s41598-025-86309-z (PMC11748720; doi:10.1038/s41598-025-86309-z)
Supplement: Supplementary file 1 — Supplementary Information. [file 41598_2025_86309_MOESM1_ESM.docx]

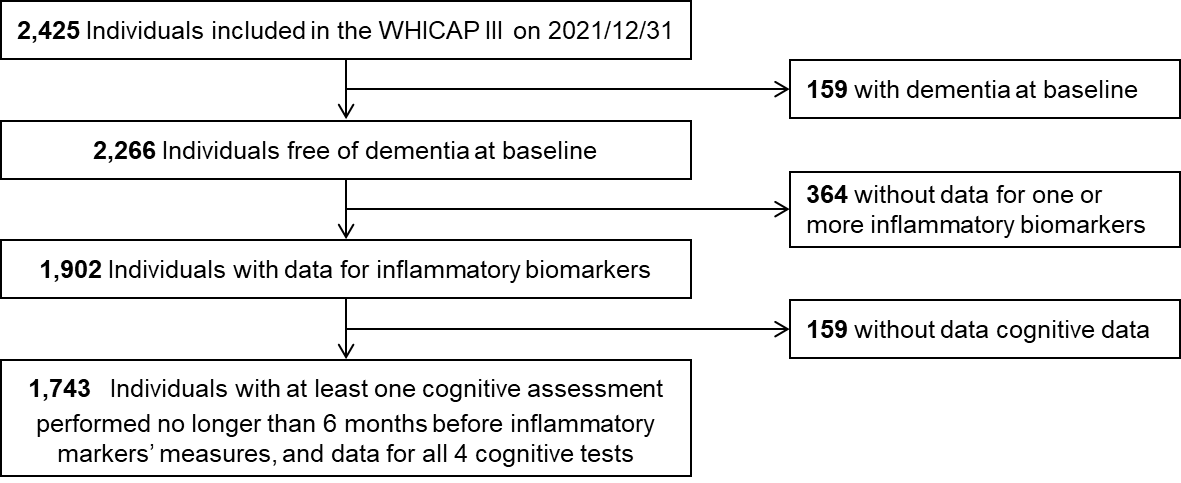


**Supplementary Figure 1. Flow chart of participants’ selection**

**
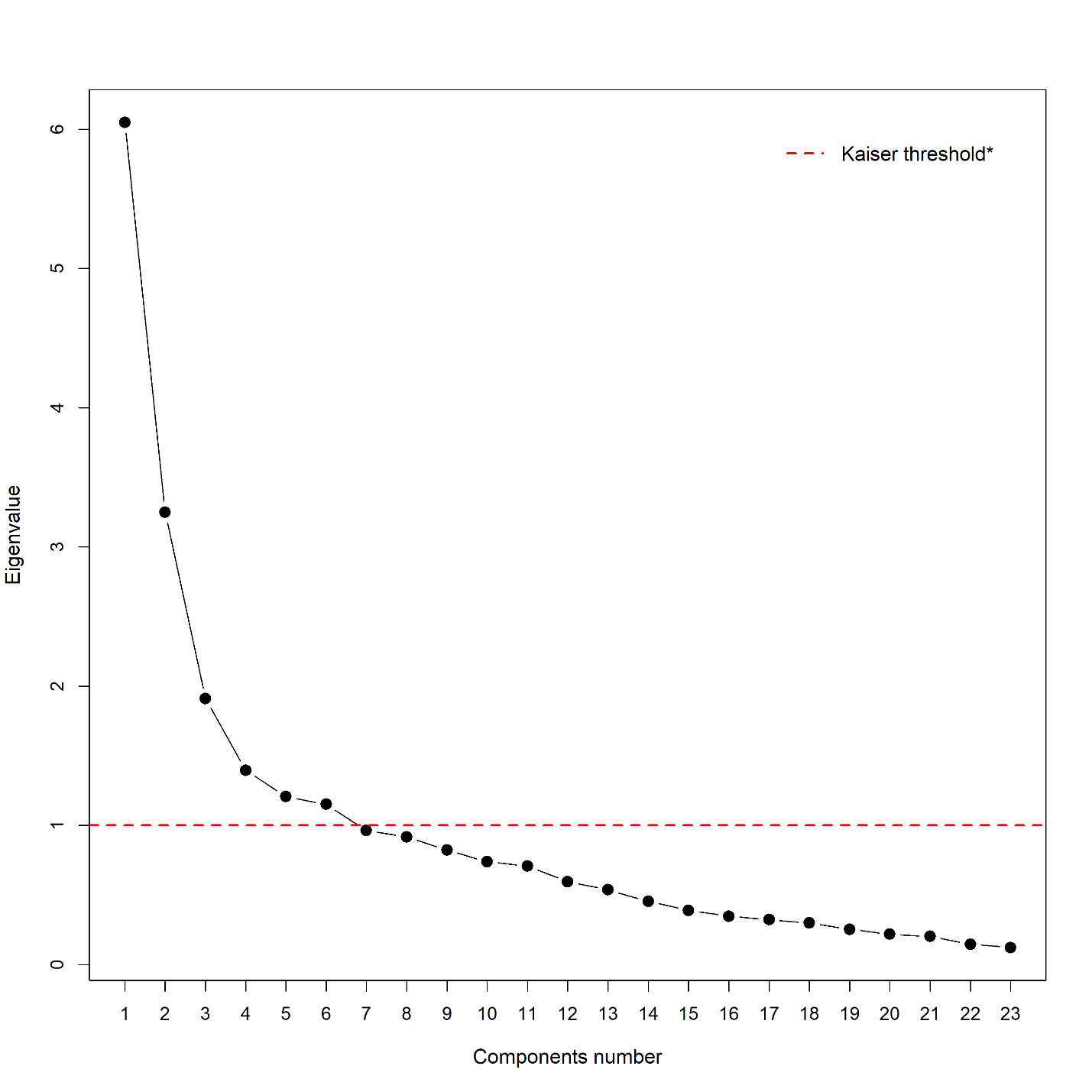
**

**Supplementary Figure 2. Scree-plot associated with the Principal Component Analysis characterizing inflammatory profiles from 23 inflammatory biomarkers, WHICAP (n = 1,743)**

* Kaiser threshold for component selection suggest keeping the 6 first components with an eigenvalue >1.

**
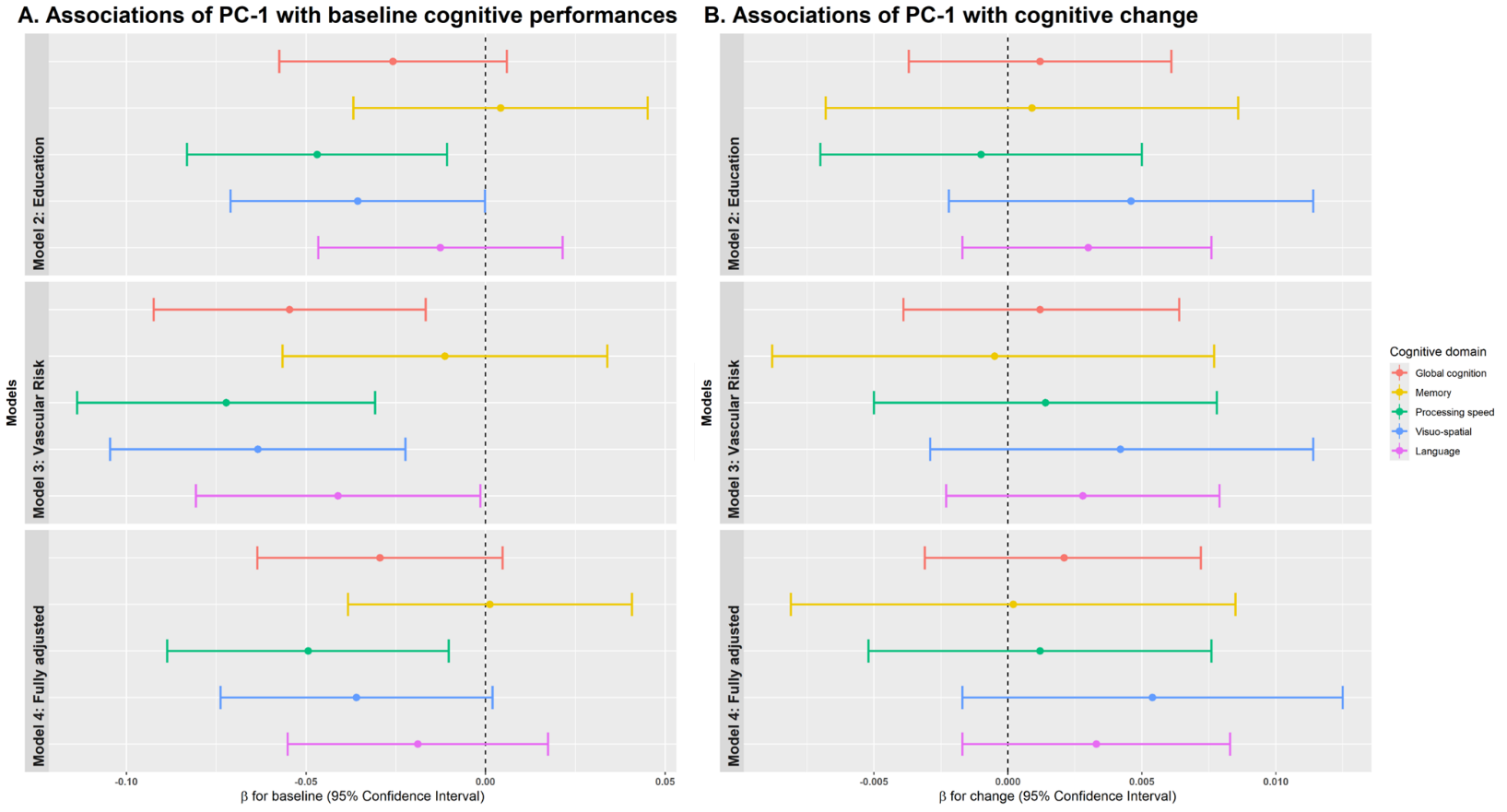
**

**Supplementary Figure 3. Association of PC-1 pro-inflammatory score with cognition, estimated by linear mixed models, WHICAP (n = 1,743)**

The figure displays the effect estimates for the associations of continuous PC-1 score with cognitive z-scores at baseline (Panel A) and cognitive decline (Panel B) over the 12-year follow-up. The trajectories of change in cognition were estimated using linear mixed models across up to 6 repeated neurocognitive examinations. Models consider a linear function of time, with corresponding random effect, and include an intercept representing the cognitive z-score at baseline (and corresponding random effect), PC-1 pro-inflammatory score (continuous, standardized), covariates, and their interactions with time.

All models were adjusted for age, gender, race/ethnicity, status for ɛ4 allele of the apolipoprotein E gene, and indicator for first cognitive assessment (Model 1). Model 2 was additionally adjusted for years of education. Model 3 was adjusted for Model 1, and body mass index, smoking status, and vascular comorbidity score. Model 4 was fully adjusted for all the above covariates and the use of anti-inflammatory medication.

Models were run on participants without missing data, sample size were n = 1,738 for Model 2, n = 1,521 for Model 3, and n = 1,517 for Model 4.

The estimate for baseline association (Panel A) is the coefficient for the PC-1 pro-inflammatory score variable term; and the estimate for cognitive decline (Panel B) is the coefficient for the PC-1 score-by-time interaction term. Effect estimates (β coefficients and 95% confidence intervals) are reported for 1-SD increase in PC-1 pro-inflammatory profile.

**
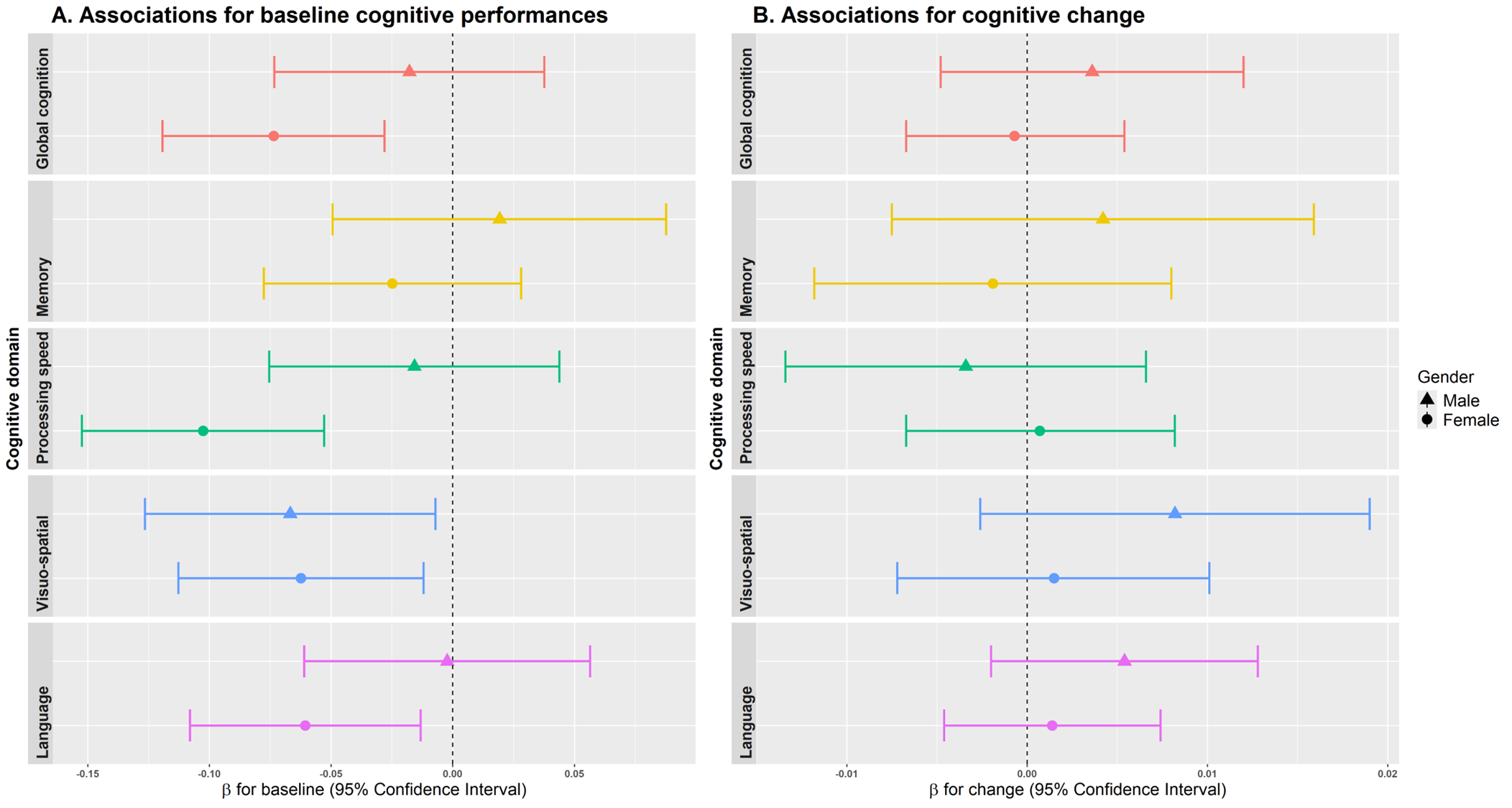
**

**Supplementary Figure 4. Associations of PC-1 pro-inflammatory score with cognition by gender, estimated by linear mixed models, WHICAP (n = 1,743)**

The figure presents the β coefficients and 95% confidence intervals for the gender-specific associations of continuous PC-1 score with cognitive z-scores at baseline (Panel A) and cognitive decline (Panel B) over the 12-year follow-up. The trajectories of change in cognition were estimated, separately for men (n = 1,589) and women (n = 3,271), using linear mixed models across up to 6 repeated neurocognitive examinations. Models consider a linear function of time, with corresponding random effect, and include an intercept representing the cognitive z-score at baseline (and corresponding random effect), PC-1 pro-inflammatory score (continuous, standardized), covariates (age, race/ethnicity, status for ɛ4 allele of the apolipoprotein E gene, and indicator for first cognitive assessment), and their interactions with time. The estimate for baseline association (Panel A) is the coefficient for the PC-1 pro-inflammatory score variable term; and the estimate for cognitive decline (Panel B) is the coefficient for the PC-1 score-by-time interaction term. Effect estimates are reported for 1-SD increase in PC-1 pro-inflammatory profile.

**
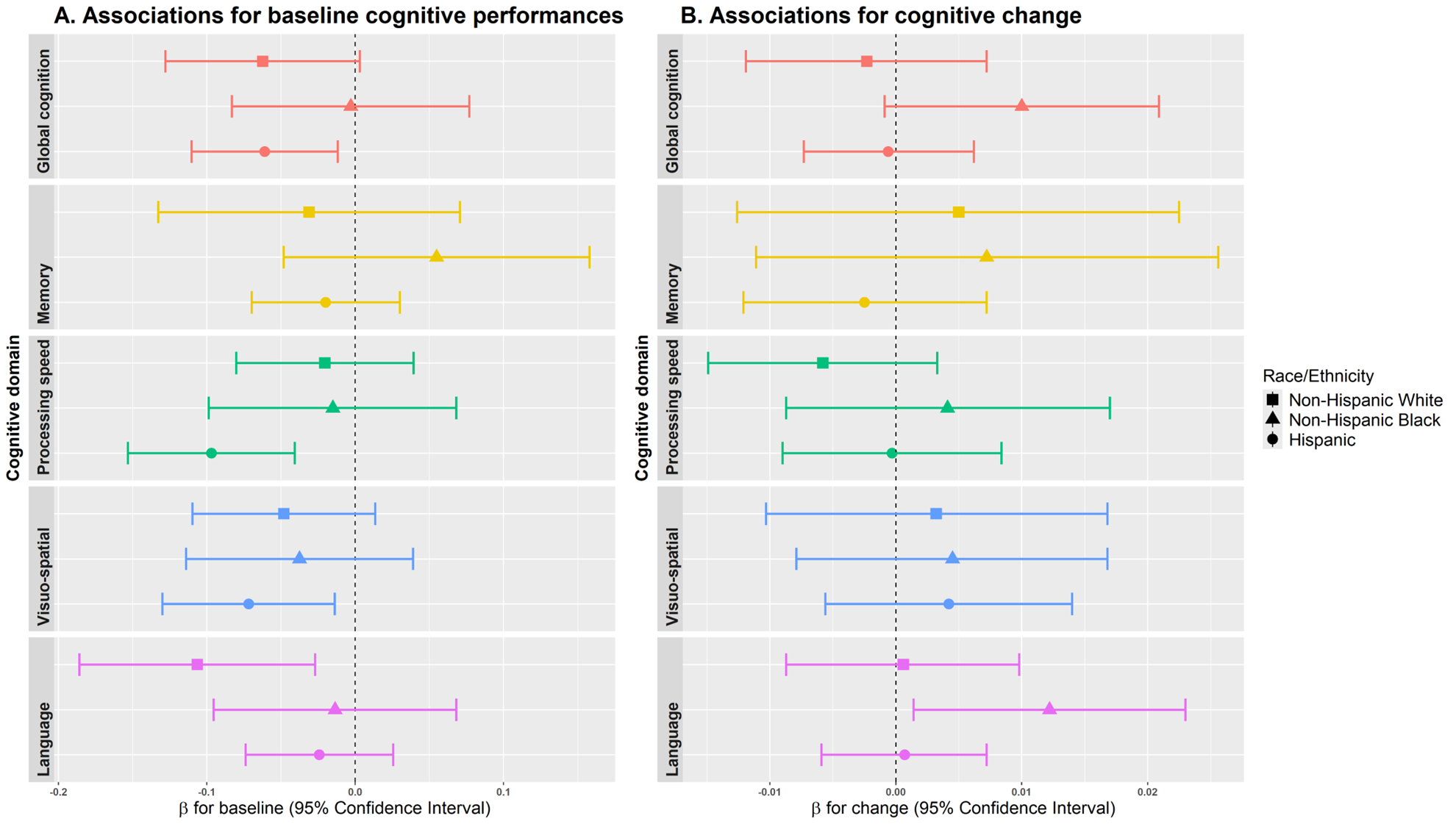
**

**Supplementary Figure 5. Associations of PC-1 pro-inflammatory score with cognition by race/ethnicity, estimated by linear mixed models, WHICAP (n = 1,743)**

The figure presents the β coefficients and 95% confidence intervals for the race/ethnicity-specific associations of continuous PC-1 score with cognitive z-scores at baseline (Panel A) and cognitive decline (Panel B) over the 12-year follow-up. The trajectories of change in cognition were estimated, separately for Non-Hispanic Whites (n = 1,165), Hispanics (n = 2,291) and Non-Hispanic Blacks (n = 1,315), using linear mixed models across up to 6 repeated neurocognitive examinations. Models consider a linear function of time, with corresponding random effect, and include an intercept representing the cognitive z-score at baseline (and corresponding random effect), PC-1 pro-inflammatory score (continuous, standardized), covariates (age, gender, status for ɛ4 allele of the apolipoprotein E gene, and indicator for first cognitive assessment), and their interactions with time. The estimate for baseline association (Panel A) is the coefficient for the PC-1 pro-inflammatory score variable term; and the estimate for cognitive decline (Panel B) is the coefficient for the PC-1 score-by-time interaction term. Effect estimates are reported for 1-SD increase in PC-1 pro-inflammatory profile.

**
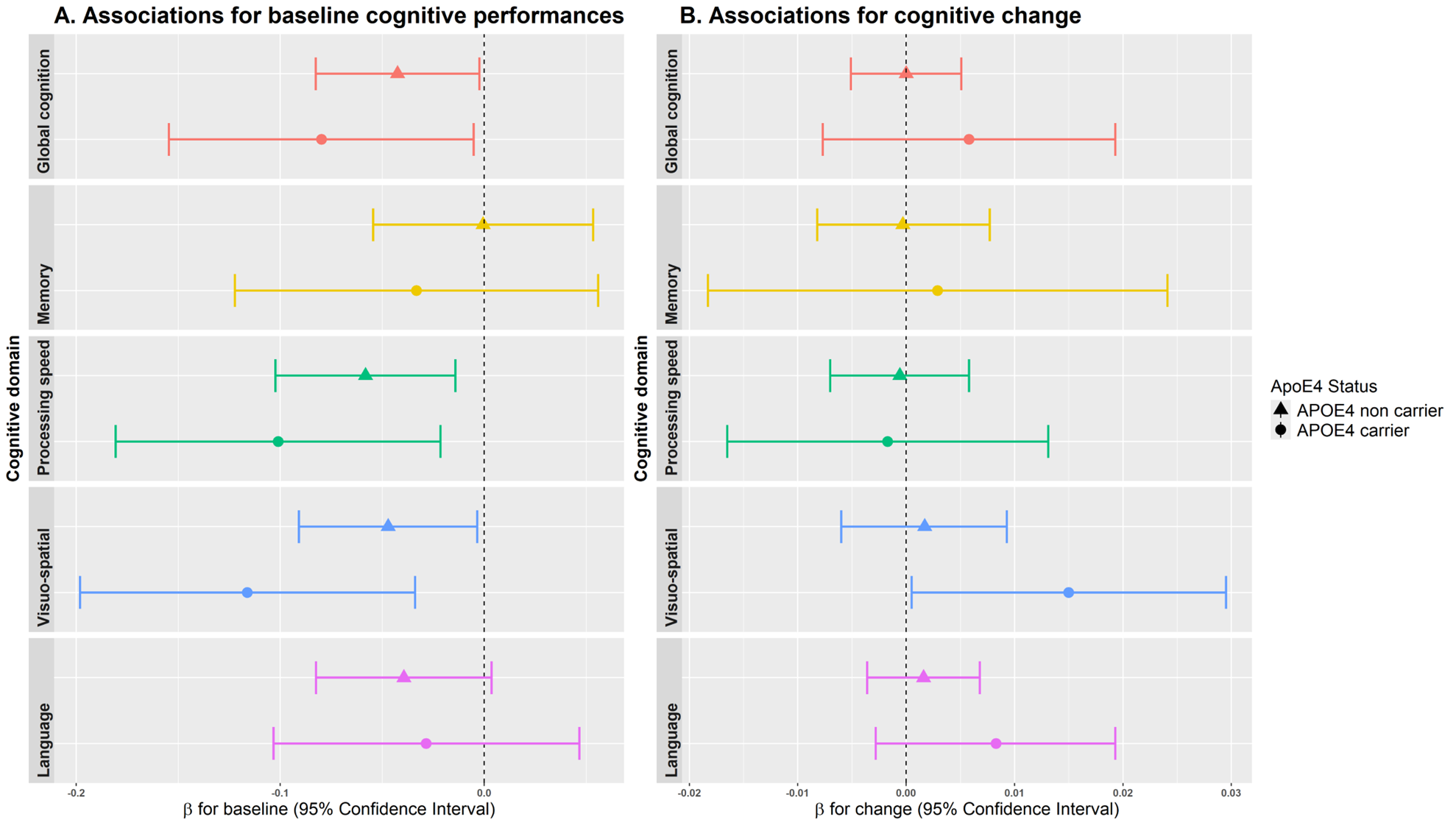
**

**Supplementary Figure 6. Associations of PC-1 pro-inflammatory score with cognition by *APOE*ɛ4 carrier status, estimated by linear mixed models, WHICAP (n = 1,743)**

The figure presents the β coefficients and 95% confidence intervals for the gender-specific associations of continuous PC-1 score with cognitive z-scores at baseline (Panel A) and cognitive decline (Panel B) over the 12-year follow-up. The trajectories of change in cognition were estimated, separately for carrier (n = 1,268) and non-carriers (n = 3,592) of the ɛ4 allele of the apolipoprotein E [*APOE*ɛ4] gene, using linear mixed models across up to 6 repeated neurocognitive examinations. Models consider a linear function of time, with corresponding random effect, and include an intercept representing the cognitive z-score at baseline (and corresponding random effect), PC-1 pro-inflammatory score (continuous, standardized), covariates (age, gender, race/ethnicity, and indicator for first cognitive assessment), and their interactions with time. The estimate for baseline association (Panel A) is the coefficient for the PC-1 pro-inflammatory score variable term; and the estimate for cognitive decline (Panel B) is the coefficient for the PC-1 score-by-time interaction term. Effect estimates are reported for 1-SD increase in PC-1 pro-inflammatory profile.

**s
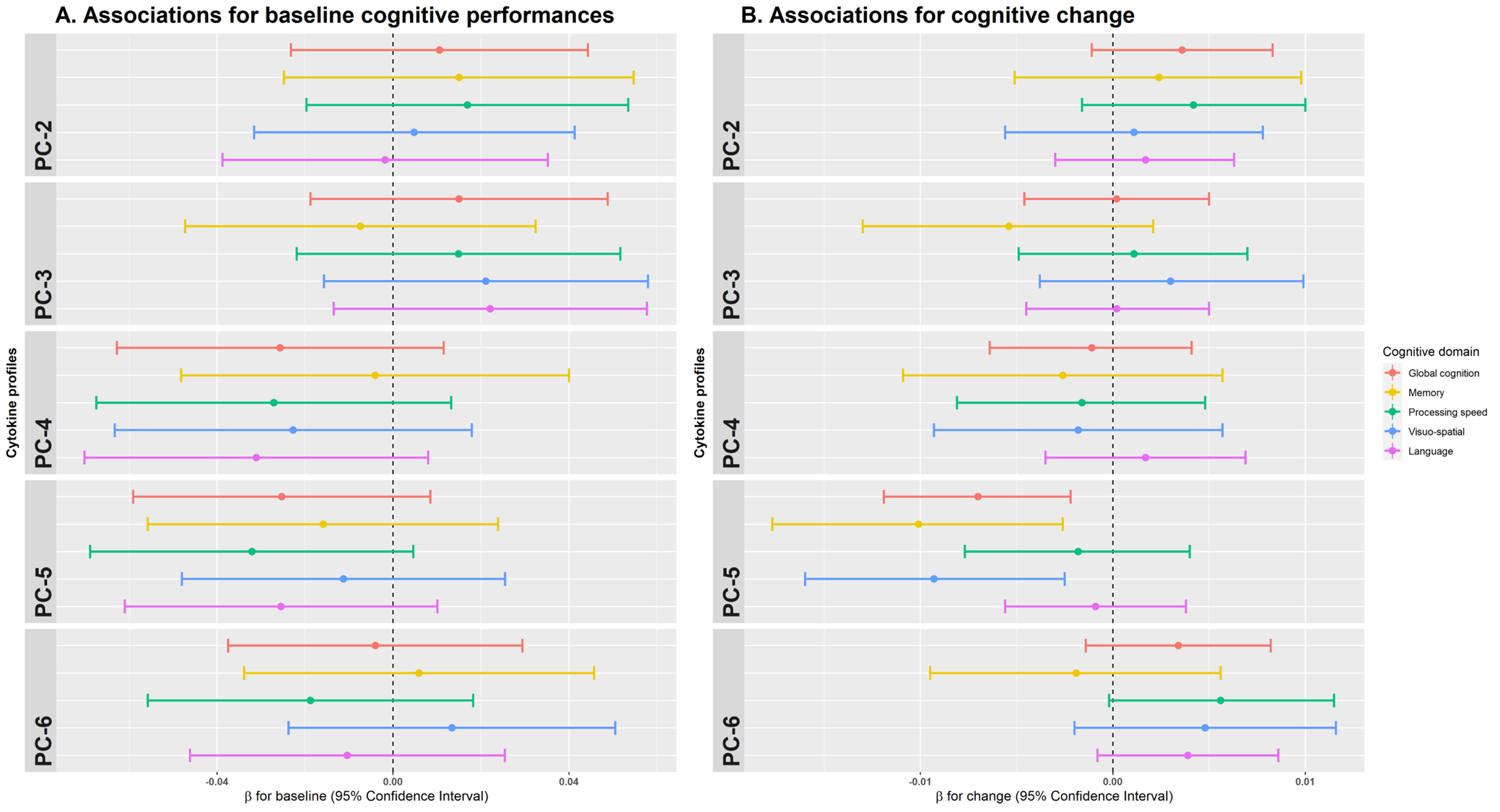
**

**Supplementary Figure 7. Associations of Principal Component scores with cognition, estimated by linear mixed models, WHICAP (n = 1,743)**

The figure displays the effect estimates for the associations of continuous PC scores with cognitive z-scores at baseline (Panel A) and cognitive decline (Panel B) over the 12-year follow-up. The trajectories of change in cognition were estimated using linear mixed models across up to 6 repeated neurocognitive examinations. Models consider a linear function of time, with corresponding random effect, and include an intercept representing the cognitive z-score at baseline (and corresponding random effect), PC scores (continuous, standardized), covariates (age, gender, race/ethnicity, status for ɛ4 allele of the apolipoprotein E gene, and indicator for first cognitive assessment), and their interactions with time. The estimate for baseline association (Panel A) is the coefficient for the PC scores variable term; and the estimate for cognitive decline (Panel B) is the coefficient for the PC score-by-time interaction term. Effect estimates (β coefficients and 95% confidence intervals) are reported for 1-SD increase in PC profile scores.
